# Immunogenicity of the Envelope Surface Unit of Human Endogenous Retrovirus K18 in Mice

**DOI:** 10.3390/ijms23158330

**Published:** 2022-07-28

**Authors:** Victoria Ilse, Rebekka Scholz, Michael Wermann, Marcel Naumann, Martin S. Staege, Steffen Roßner, Holger Cynis

**Affiliations:** 1 Department of Drug Design and Target Validation, Fraunhofer Institute for Cell Therapy and Immunology, Weinbergweg 22, 06120 Halle, Germany; victoria.ilse@izi.fraunhofer.de (V.I.); rebekka.scholz@dzne.de (R.S.); michael.wermann@izi.fraunhofer.de (M.W.); marcel.naumann@izi.fraunhofer.de (M.N.); 2 Department of Surgical and Conservative Pediatrics and Adolescent Medicine, Medical Faculty, Martin Luther University Halle-Wittenberg, Ernst-Grube-Str. 40, 06097 Halle, Germany; martin.staege@uk-halle.de; 3 Paul Flechsig Institute for Brain Research, Leipzig University, Liebigstraße 19, 04103 Leipzig, Germany; steffen.rossner@medizin.uni-leipzig.de

**Keywords:** human endogenous retrovirus, retroviral envelope, HERV-K18, multiple sclerosis, experimental autoimmune encephalomyelitis

## Abstract

The triggers for the development of multiple sclerosis (MS) have not been fully understood to date. One hypothesis proposes a viral etiology. Interestingly, viral proteins from human endogenous retroviruses (HERVs) may play a role in the pathogenesis of MS. Allelic variants of the HERV-K18 env gene represent a genetic risk factor for MS, and the envelope protein is considered to be an Epstein–Barr virus-trans-activated superantigen. To further specify a possible role for HERV-K18 in MS, the present study examined the immunogenicity of the purified surface unit (SU). HERV-K18(SU) induced envelope-specific plasma IgG in immunized mice and triggered proliferation of T cells isolated from these mice. It did not trigger phenotypic changes in a mouse model of experimental autoimmune encephalomyelitis. Further studies are needed to investigate the underlying mechanisms of HERV-K18 interaction with immune system regulators in more detail.

## 1. Introduction

Approximately 8% of the human genome consists of human endogenous retroviral (HERV) elements. They originated from putative exogenous retroviral infections of the germ line at various time points during human evolution [1]. Most of these viral DNA sequences are mutated or epigenetically suppressed, such that no infectious HERV has yet been identified [2]. Nevertheless, intact open reading frames have persisted in the genome, which can be activated epigenetically or by environmental factors in genetically susceptible individuals [3,4,5,6]. As a consequence, viral RNA transcripts and/or proteins are formed, which might possess a function in the human body [7]. As an example, syncytin-1, the envelope protein of HERV-W1, is involved in placentation [8]. Other HERV transcripts have been found to be increased in pathologically altered human tissues or in the blood and plasma of patients, which is why HERVs are associated with certain cancers [9,10] and neurologic diseases, such as amyotrophic lateral sclerosis [11] and multiple sclerosis (MS) [12,13]. In particular, the envelope protein of the multiple sclerosis-associated retrovirus (MSRV), which belongs to the HERV-W family, is highly expressed in active MS lesions [14,15], exhibits proinflammatory and neurotoxic effects [16,17], and inhibits oligodendrocyte precursor cell differentiation in vitro [18]. Although no causal link between HERV expression and the development of MS has yet been established, the therapeutic antibody temelimab, specific to the HERV-W envelope protein, has been tested in relapsing-remitting MS (the phase 2b clinical studies CHANGE-MS and ANGEL-MS, ClinicalTrials.gov identifiers: NCT02782858 and NCT03239860, respectively) [19]. However, no reduction in the total numbers of gadolinium-enhancing lesions and clinically apparent relapses were recorded [20,21]. Non-significant reductions in brain atrophy and magnetization transfer ratio decrease indicate potential benefits in neurodegeneration [21].

In addition to HERV-W, the association of other HERVs, such as HERV-K, with MS has been suggested. The HERV-K human MMTV-like (HML-2) family shares sequence similarity with mouse mammary tumor virus (MMTV) and is named based on the primer binding site for Lys-tRNA [22]. Members of most HERV families, and particularly the HERV-K family, are polymorphic, meaning that not all individuals have the same set of retroviruses at the same genomic location [23,24]. For example, HERV-K113 is present in a maximum of 30% of all individuals, with high variation depending on geographic the region of origin [25,26]. Such interindividual differences may affect disease susceptibility, as is known for HERV-K18, where homozygous carriers of the K18.3 allele have a higher disease risk for MS compared with carriers of the K18.1 and K18.2 alleles [27,28]. However, such genetic associations cannot explain the pathogenesis of MS without experimental proof of the corresponding biological activity; e.g., in animal models. Furthermore, linkage disequilibria between the identified loci and other disease-modifying gene loci might be involved in the reported associations.

While HERVs are usually transcriptionally silent, they can be activated by environmental factors, such as virus infections. For example, Epstein–Barr virus (EBV) transactivates HERV-K18 in resting B-lymphocytes through binding of CD21 [29]. Additionally, human herpesvirus 6 (HHV-6) induces transcriptional activation of the HERV-K18 envelope in cells from peripheral blood [30,31]. Infection with human herpes viruses has been suggested to contribute to the development of MS (reviewed in [32] (HHV-6) and [33] (EBV)). A recent study shows that EBV infection appears to be the primary cause of MS development [34]. However, protein expression of HERV-K18 in MS has not been described so far. Transactivation of a putative superantigen encoded by HERV-K18 [35,36,37,38] may provide the missing link between viral infection and inflammatory events seen in MS. Based on (I) the genetic association of HERV-K18 with MS and (II) the herpesvirus trans-activation of HERV-K18, the aim of this study was to further characterize the immunogenic potential of HERV-K18 proteins. Therefore, the recombinant SU of HERV-K18 was studied for its ability to induce proliferation of T cells, to elicit specific IgG titers in immunized mice, and to provoke symptoms of experimental autoimmune encephalomyelitis (EAE), the classic mouse model for MS, in challenged mice. We used the SU instead of the whole protein because we were focused on studying the immune interaction determined by the SU rather than the transmembrane unit that anchors the protein in the membrane and is responsible for the fusion of the viral membrane with the host membrane.

## 2. Results

### 2.1. HERV-K18(SU) Was Purified from E. coli Inclusion Bodies

**Figure 1:**
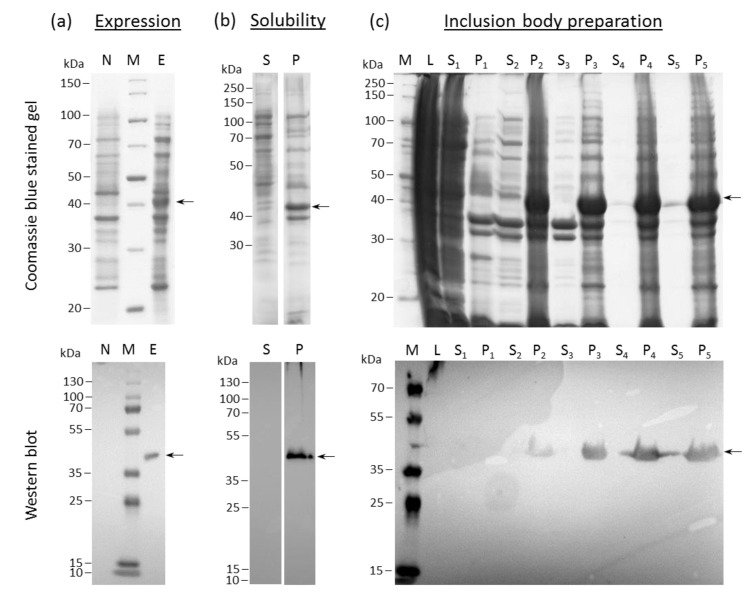
Coomassie brilliant blue-stained SDS-PAGE (top) and anti-His6 Western blot analysis (bottom) illustrating the expression, solubility, and inclusion-body preparation of K18(SU) from *E. coli* Rosetta (DE3). (**a**) Specific protein expression (E) in transformed bacterial cultures induced with 1 mM IPTG can be observed in comparison to non-induced control samples (N). (**b**) Analysis of pellet (P) and supernatant (S) after cell lysis and centrifugation revealed that K18(SU) was expressed in insoluble form. (**c**) Enrichment of K18(SU) through sequential washing steps (S: supernatants, P: pellets after centrifugation) during inclusion-body preparation can be observed in comparison to the bacterial lysate (L). M: Protein ladder (Coomassie blue-stained gels) or molecular weight marker standards (Western blots). Arrows point to the target protein.

The expression of HERV-K18(SU) was assessed through the visualization of an additional protein band in lysates of induced cultures compared with a non-induced control in reducing SDS-PAGE (Figure 1a, top). As HERV-K18(SU) was fused to a C-terminal hexa histidine-tag, Western blot analysis using an anti-hexa histidine-tag antibody could confirm this result (Figure 1a, bottom). The observed molecular weight of the recombinant protein corresponded to a theoretical molecular mass of 42 kDa. HERV-K18(SU) was present in the insoluble fraction (Figure 1b). This is not surprising since bacteria are not able to generate the N-glycans of viral envelope proteins, which may lead to misfolding and inclusion-body formation. Since inclusion bodies accumulate large amounts of the target protein, their preparation was the first purification step for the removal of cellular proteins. Inclusion bodies were solubilized in 6 M guanidine hydrochloride and subjected to consecutive washes with buffers containing Triton-X 100. In this way, membrane proteins could be separated from the target protein, as made visible by bands in the supernatants of Coomassie brilliant blue stained polyacrylamide gels (Figure 1c, lanes S1–S3). Two additional washes without Triton-X 100 were performed to remove the detergent (Figure 1c, lanes S_4_–P_5_). Although HERV-K18(SU) could be enriched in the pellet fractions, impurities from other proteins required further purification.

**Figure 2:**
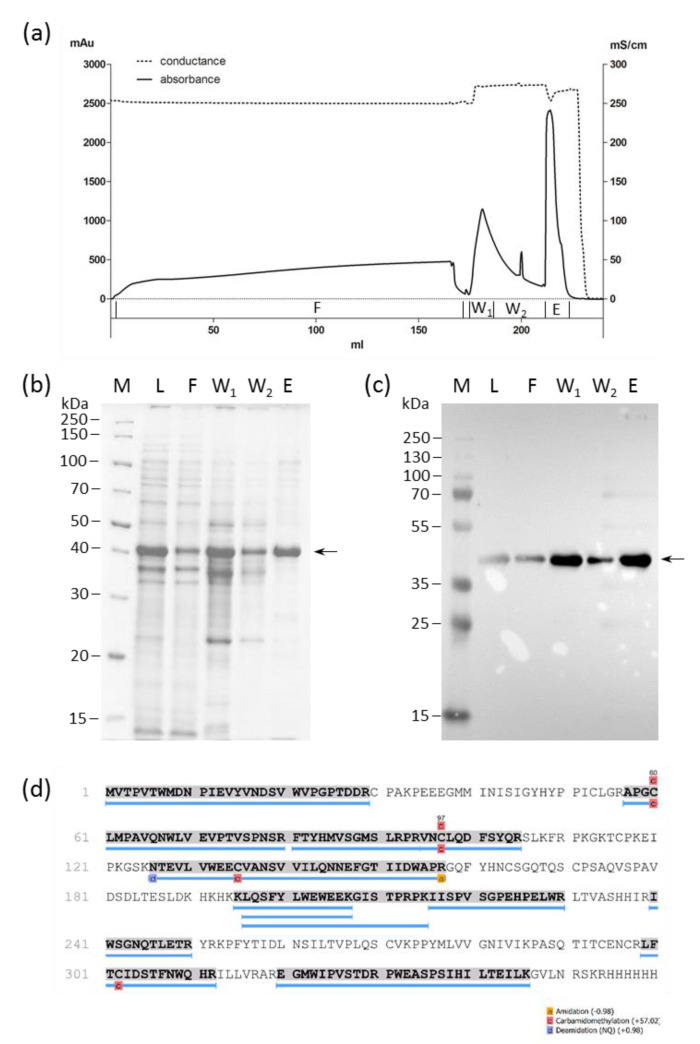
Purification of HERV-K18(SU) from bacterial inclusion bodies using nickel nitrilotriacetic acid (NTA) chromatography. (**a**) The chromatogram shows absorbance at 280 nm and electric conduction, as well as the fractions collected during the purification process (F: flow through; W1 and W2: wash with 25 mM imidazole; E: elution with 250 mM imidazole). (**b**) Coomassie brilliant blue-stained SDS-PAGE of the load (L) and the fractions depicted in (**a**). M: protein ladder. (**c**) Anti-His6 Western blot of the load (L) and the fractions depicted in (**a**). M: molecular weight marker standard. Arrows point to the target protein. (**d**) Mass spectrometric analysis of the elution fraction. The protein band corresponding to the target protein was excised from the polyacrylamide gel in the elution fraction, trypsin-digested and analyzed with mass spectrometry for matches (blue lines) with the template sequence. The sequence coverage was 55%.

Nickel nitrilotriacetic acid (Ni-NTA) chromatography under denaturing conditions resulted in 150 mg HERV-K18(SU) (results not shown). However, because flow through and wash fractions also contained the target protein, they were subsequently pooled and purified again to increase the yield. The purification process and the analysis of the fractions obtained are shown in Figure 2. The final purified protein yield obtained was >39.6 mg per liter bacterial culture with a purity of 76%, according to SDS-gel analysis. A mass spectrometric analysis of the gel band belonging to the elution fraction confirmed the identity and integrity of HERV-K18(SU) (Figure 2d).

### 2.2. Recombinant HERV-K18(SU) Triggered Humoral and Cellular Immune Responses

**Figure 3:**
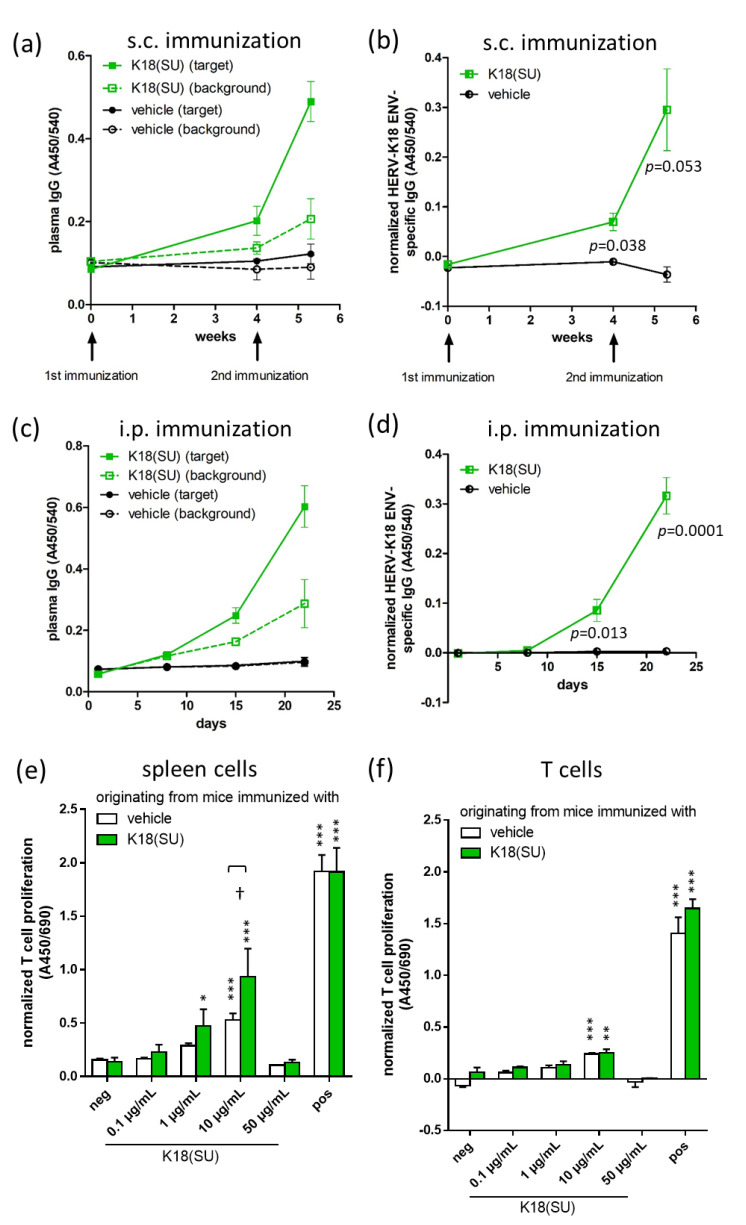
Antibody and T-cell response to recombinant HERV-K18(SU). (**a**,**b**) Six week old female Balb/c mice received two subcutaneous immunizations (indicated by arrows) with 50 µg HERV-K18(SU) (*n* = 3) or a vehicle (*n* = 2) in IFA with a four-week interval. Plasma IgG titers were analyzed at three time points using HERV-K18 Env-specific ELISA. As ELISA plates could not be coated with insoluble recombinant HERV-K18(SU), cell extracts that did (target) or did not (background) contain HERV-K18 Env were used instead. Graphs in (**a**,**b**) show original absorbance data and the background subtracted values, respectively. Representative results of one of two independent experiments are shown as the mean ± SEM. Statistics: unpaired *t*-test (K18(SU) versus vehicle). (**c**,**d**) Balb/c mice (*n* = 5) were immunized intraperitoneally with 10 µg HERV-K18(SU) daily for 3 × 5 days. Plasma IgG titers were analyzed using HERV-K18 Env-specific ELISA as described above. Graphs in (**c**) and (**d**) show original absorbance data and the background subtracted values, respectively. Statistics: unpaired *t*-test (K18(SU) versus vehicle). (**e**,**f**) Isolated splenocytes (**e**) or MACS-sorted T cells (**f**) from immunized mice were isolated and stimulated with different amounts of HERV-K18(SU), buffer (neg), or dynabeads (pos) for 48 h. Thereafter, T-cell proliferation was determined using BrdU ELISA. Bars show means ± SEM (each *n* = 3). Statistics: one-way ANOVA with Dunnett’s post hoc test (neg versus all other groups), * *p* < 0.05, ** *p* < 0.01, *** *p* < 0.001. Unpaired *t*-test, † < 0.05.

To analyze the antigenicity of recombinant HERV-K18(SU), six-week-old female Balb/cAnNRj mice were immunized two times with an interval of four weeks with an emulsion containing 50 µg recombinant HERV-K18(SU) and incomplete Freund’s adjuvant (IFA). HERV-K18 Env-specific IgG titers increased compared to the vehicle control four weeks after the first immunization and increased even more after the second immunization (Figure 3a,b and Supplementary Figure S1). To further study the K18-specific humoral immune response without the immunostimulatory effect of IFA, female Balb/c mice were injected intraperitoneally with 10 µg protein per day for 3 × 5 consecutive days, which was followed by determination of plasma IgG antibody titers towards HERV-K18 Env. A significant increase in the HERV-K18 Env-specific IgG titer was observed from day 15 onwards (Figure 3c,d). To further study the cellular immune response towards HERV-K18(SU), splenocytes of immunized mice were stimulated with HERV-K18(SU) for 48 h in vitro. Stimulation with buffer and Dynabeads^®^ Mouse T-Activator CD3/CD28 served as negative and positive controls, respectively. Then, T-cell proliferation was measured using BrdU incorporation ELISA. The results showed that T-cell proliferation increased with higher doses of HERV-K18(SU), becoming significant at 1 µg/mL HERV-K18(SU) in comparison to the negative control (Figure 3e). High concentrations of the envelope protein (50 µg/mL) seemed to be cytotoxic since proliferation returned to base line. Cells isolated from mice immunized with the SU (Figure 3e, green bars) showed higher proliferation compared to mice immunized with the vehicle (Figure 3e, white bars). Interestingly, a HERV-K18(SU) dose-dependent increase in T cell proliferation was observed even in MACS-isolated T cells containing no antigen-presenting cells (APCs), although the effect was not as high as observed in splenocytes and was possibly due to residual APCs in the preparation. In summary, recombinant HERV-K18(SU) was found to induce both a humoral and cellular immune response.

### 2.3. HERV-K18(SU) Did Not Induce Phenotypic Changes in EAE

**Figure 4:**
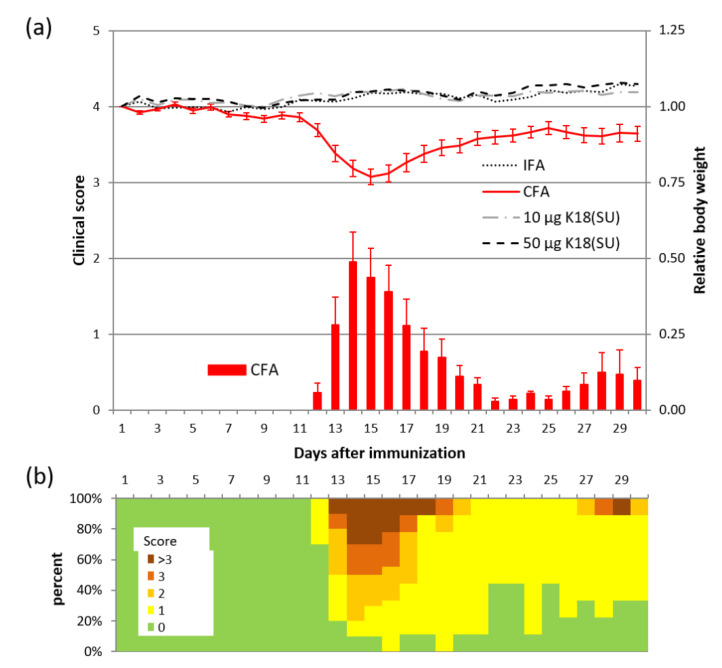
HERV-K18(SU) does not provoke an immune reaction in a model of EAE. (**a**) Clinical score (bar graph) and relative body weight (line graph) over 30 days for mice immunized with an emulsion containing either Freund’s complete adjuvant (CFA, *n* = 10), Freund’s incomplete adjuvant (IFA, *n* = 5), or Freund’s incomplete adjuvant and 10 µg or 50 µg K18(SU) (*n* = 10, respectively). Data are represented as means with SE, except for the mean relative body weight of the groups receiving IFA, 10 µg K18(SU) and 50 µg K18(SU) for reasons of clarity. (**b**) Percentage distribution of clinical scores for CFA-immunized mice. Note that one mouse showed a biphasic disease course with a second peak at day 29.

**Figure 5:**
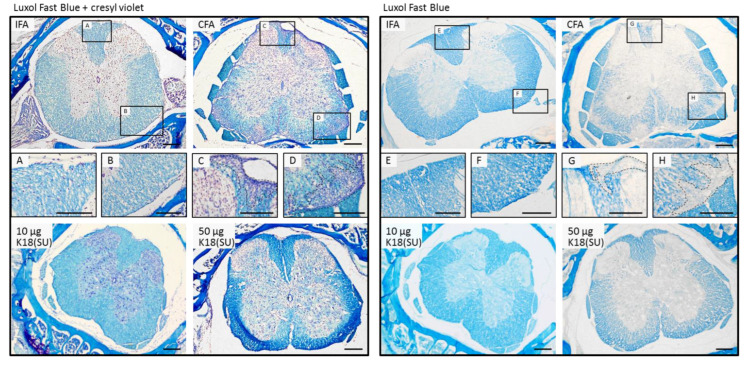
Luxol fast blue staining of representative cross-sections of the spinal cord of immunized mice for visualization of myelin sheaths. Treatment is indicated in the corner of each large image. Counter-stains with cresyl violet acetate shown in the left panel indicate staining of cell nuclei. (**A**–**H**) Magnifications of the areas labeled in the IFA and CFA sections to show intact white matter (**A**,**B**,**E**,**F**) and demyelinated areas (**C**,**D**,**G**,**H**) with putative infiltrated immune cells ((**C**,**D**,**G**,**H**)**,** dotted lines). Scale bar: 200 µm.

In order to investigate the immune-stimulatory potential of HERV-K18(SU) in the MS context in more detail, we chose the EAE model for our studies. Typically, EAE mice receive an immunization with myelin antigens in combination with complete Freund’s adjuvant, which contains mycobacteria, to induce a strong immune response towards the antigen. This leads to demyelination of the nerve processes in the CNS, as reflected by paresis and paralysis of the tail and extremities in mice. In our experiments, mycobacteria were completely replaced by HERV-K18(SU) to test whether it was equally capable of inducing EAE-type symptomatology in mice. The positive control group of mice (*n* = 10) was immunized with complete Freund’s adjuvant (CFA), which contained desiccated mycobacterium tuberculosis, to induce symptoms as seen in classic EAE (positive control). Two different doses (10 µg and 50 µg; *n* = 10, respectively) of purified HERV-K18(SU) were used in place of mycobacteria to study the immune-stimulatory effects of the viral envelope. Mice immunized with incomplete Freund’s adjuvant (IFA) (*n* = 5) served as a negative control. All CFA-immunized mice (incidence 100%) developed symptoms, including limp tails and paresis or paralysis of hind limbs, with a mean latency of 13 days and a mean maximum clinical score of 1.95 at day 14 (Figure 4a). The increase in the clinical score was accompanied by a reduction in body weight to a minimum of 77% of the initial weight on average. A monophasic course of disease with remission of symptoms was observed in all but one mouse, which showed a biphasic course with a clinical score of 3 on day 29, producing the biphasic course with a second maximum at the end of the experiment (Figure 4a,b). None of the mice in the other groups developed a clinical score different from zero or lost body weight significantly. Histological examinations of spines supported these observations. Demyelination of the white matter at the rim of the spinal cord accompanied by infiltration of putative immune cells was observed in CFA-immunized mice, whereas no impairment of myelin sheaths was found in HERV-K18(SU)-immunized mice (Figure 5). In conclusion, HERV-K18(SU) purified from *E. coli* lysates did not induce EAE-like phenotypic changes in immunized mice.

## 3. Discussion

Once activated by intrinsic or environmental triggers, such as virus infection, HERV can produce viral proteins whose functions are largely unknown. Due to their similarity to exogenous retroviral glycoproteins, HERV envelope proteins feature a high immunogenic potential and may induce inflammatory events in the human body. In this context, the present study demonstrated that the SU of HERV-K18 triggered the proliferation of T cells in primary mouse spleenocytes and induced a humoral immune response in immunized mice.

As a prerequisite for functional studies, the SU of HERV-K18 was expressed in *E. coli* and purified using immobilized metal ion affinity chromatography (Figure 1). Previously, the expression of other HERV envelope proteins has been successfully achieved in *E. coli* by other groups [39,40]. As such, the glycoproteins were always expressed insolubly due to the inability of *E. coli* to form post-translational modifications, such as glycosylation. Nevertheless, these proteins showed biological activity in functional assays in ours and other studies [17,18,40,41,42].

We could show that recombinant HERV-K18(SU) induced envelope-specific plasma IgG in immunized mice (Figure 3). The immune response was independent of the use of an adjuvant, making HERV-K18(SU) immunogenic per se. Moreover, stimulation of mouse splenocytes with HERV-K18(SU) in vitro resulted in a dose-dependent and significant increase in T-cell proliferation, which was highest at 10 µg/mL (Figure 3). These observations indicate that HERV-K18 envelope protein is recognized in the immunized mice as an antigen. Per its definition, HERV-K18 is a xeno-antigen in mice. Whether this protein is also recognized as an antigen in human individuals might depend on genetic (e.g., presence of the corresponding HERV-K18 allele in the genome of the individual) as well as non-genetic (e.g., the former contact with cross-reacting exogenous antigens) factors.

Other researchers have proposed HERV-K18 to be a superantigen. In this context, HERV-K18 (formerly named IDDMK_1,2_22)-expressing cells activated Vβ T-lymphocytes [35,36]. Similar effects could be demonstrated in cell culture experiments, where T-cell subsets carrying the T-cell receptor Vβ13 were activated by HERV-K18 envelope-expressing murine B cells [37]. The same group showed a clonal reduction of peripheral Vβ3+ T cells in HERV-K18 transgenic mice, demonstrating the reactivity of this T-cell subset towards the HERV-K18 envelope [38]. However, these findings have been questioned by other investigators. For example, Lapatschek et al. [43] could not reproduce previous findings because neither murine nor human T cells were stimulated in the presence of IDDMK_1,2_22. Similarly, independent investigations could not detect activation of murine T-cell hybridomas by IDDMK_1,2_22 [44]. The results of the present study do not allow any conclusions to be drawn about the super-antigenic properties of HERV-K18. For this, T-cell subpopulations would have to be studied in more detail. In addition, whether HERV-K18 has superantigenic activity for human T or B cells has to be investigated.

To bridge the gap from a general immunogenic effect of HERV-K18 to a possible involvement in autoimmune diseases, as discussed in the literature [27,28,45,46], we used the EAE model. Previous work from H. Perron and colleagues [39] showed that the full-length and surface units of HERV-W were able to trigger EAE-like symptoms in mice if they were administered instead of mycobacteria. In our study, only mice immunized with mycobacteria-containing CFA showed an increase in the clinical score, as seen in classic EAE with demyelinated white matter in the spinal cord (Figure 4 and Figure 5). The mice immunized with HERV-K18(SU) showed neither clinical (clinical score) nor histological signs of an inflammatory reaction in the central nervous system (CNS), as shown by myelin staining of spinal cord sections (Figure 5).

The fact that, in contrast to the SU of HERV-W, HERV-K18(SU) injection did not trigger phenotypic changes in EAE mice might be due to methodological differences, including the genetic background of mice, the CNS antigen used, or the immunization scheme. The use of additional immune boosters, such as pertussis toxin, that impair the blood–brain barrier [39] seems to be less relevant for our experimental setting, as activated T cells should cross this barrier, as also shown by the EAE induction with CFA. On the other hand, one cannot exclude the possibility that the full-length envelope protein of HERV-K18 causes EAE-like symptoms. Furthermore, the expression of full-length HERV-K18 in eukaryotic hosts might reveal further insights regarding its immunogenicity.

The question remains concerning how the results of the current study in mice translate to humans. For HERV-K18 to exert its immunogenic effect in the human body after putative transcriptional activation, two prerequisites have to be met. The first is successful protein biosynthesis of the HERV-K18 envelope protein. Although HERV-K18 possesses a complete open reading frame for the envelope at chromosome 1q23.3, the envelope gene carries a 292 bp deletion in the pol-env boundary [47]. This leads to impaired glycosylation in transfected human cell lines and may cause improper trafficking [48], which in turn could impede interaction with immune cells. The second is missing self-tolerance towards the envelope protein. Only when HERV-K18 Env is not present as a self-antigen during T-lymphocyte maturation will it be later targeted by the immune system. There is evidence in the literature that other HERV Envs are not recognized as “self”. For example, the envelope protein of HERV-W induces pro-inflammatory cytokines in various human cells (brain endothelium, monocytes, oligodendroglial progenitor cells, peripheral blood mononuclear cells) [17,18,40,41,42].

However, if the above two conditions are met, based on the results of this study, it can be hypothesized that transcriptionally activated HERV-K18 Env promotes immune responses in the human body.

## 4. Materials and Methods

### 4.1. Cloning and Expression of HERV-K18 SU in E. coli Rosetta (DE3)

HERV-K18 envelope DNA (GenBank accession no. AF333069.1) was synthesized de novo by an external company (Genscript, Piscataway, NJ, USA). Subsequently, the SU of HERV-K18 env (bp 1-1062) was cloned in pET28a(+) (Novagen, Darmstadt, Germany) using standard procedures [49] via NcoI and NotI restriction sites and using appropriate primers (forward: ATA TCC ATG GTA ACA CCA GTC ACA TGG ATG; reverse: ATA TGC GGC CGC TCA GTG ATG GTG ATG GTG ATG TCT TTT GGA TCT ATT TAA AAC). A sequence encoding the hexa histidine-tag was added to the C-terminus. Positive clones were confirmed by restriction digestion and nucleotide sequencing. The recombinant plasmids (50 ng) were transformed into chemically competent *E. coli* Rosetta (DE3) cells (Novagen) [50]. Transformed cells were grown in 1 mL of LB medium. After 1 h of incubation at 200 rpm at 37 °C, 100 µL of the culture was spread on LB agar with 50 mg/mL of kanamycin for selection and incubated at 37 °C overnight. A colony was used to inoculate 100 mL LB medium containing kanamycin (50 mg/mL), which was incubated with shaking at 120 rpm at 37 °C overnight. The next day, 6 L TB medium (1.2% tryptone, 2.4% yeast extract, 0.5% glycerol, 0.017 M KH_2_PO_4_, 0.072 M K_2_HPO_4_) with antibiotic was inoculated with 0.5% of the pre-culture and further grown at 100 rpm at 37 °C. At an optical density measured at a wavelength of 600 nm of 1, isopropyl β-D-1-thiogalactopyranoside (IPTG) was added to a final concentration of 1 mM and the culture was further grown with shaking at 37 °C. After 2 h, the cells were collected by centrifugation at 6000× *g* at 4 °C for 30 min and stored at −20 °C.

### 4.2. Protein Solubility

Ten milliliters of induced cultures was pelleted and resuspended in one milliliter of 100 mM Tris (pH 8.0) buffer containing 200 mM NaCl and 1x cOmplete protease inhibitor cocktail (Roche, Basel, Switzerland). Cells were lysed using a Precellys 24 homogenizer (Bertin Technologies, Montigny-le-Bretonneux, France) in 2 cycles of 20 s at 6000 rpm, with cooling on ice in between. The lysate was pelleted by centrifugation at 16,000× *g* at 4 °C for 10 min. The supernatant and pellet were collected separately and analyzed in reducing SDS-PAGE.

### 4.3. Preparation and Extraction of Insoluble Proteins (Inclusion Bodies) from E. coli

The cell pellet was resuspended in 5 mL lysis buffer (100 mM Tris, pH 7.8, 1 mM EDTA) per gram wet biomass using a homogenizer (Miccra, Müllheim, Germany) and incubated with 1.5 mg lysozyme per gram wet biomass for 1 h at room temperature. Subsequently, the cells were lysed in 4 consecutive cycles with a high-pressure homogenizer Avestin Emulsiflex C5 (ATA Scientific, Taren Point, Australia) at 68.95 bar piston pressure. The cell suspension was mixed with DNase I (Applichem, Darmstadt, Germany) to a final concentration of 10 µg/mL and MgCl_2_ to a final concentration of 5 mM and incubated for 1 h at room temperature, followed by addition of 0.5 volume wash buffer I (1.5 M NaCl, 60 mM EDTA, pH 7.4, 6% (*v*/*v*) Triton X-100) and incubation for 1 h on ice. After centrifugation (11,300× *g*, 1 h, 4 °C) of the cell suspension, the resulting pellet was washed 2 times in 6 mL wash buffer II (100 mM Tris, pH 7.4, 20 mM EDTA, 500 mM NaCl, 2% (*v*/*v*) Triton X-100) per gram wet biomass of the initial cell pellet, followed by 2 washes in 6 mL wash buffer III (100 mM Tris, pH 7.4, 20 mM EDTA) per gram wet biomass. Centrifugation during all wash steps was carried out at 17,700× *g*, 30 min, 4 °C. The last step was a centrifugation step. The inclusion body pellet was stored at −20 °C until further use.

### 4.4. Purification of Proteins from Inclusion Bodies

The His_6_-tagged HERV-K18(SU) was purified using immobilized metal ion affinity chromatography. The inclusion body pellet was solubilized in 1 mL solubilization buffer (100 mM Tris, 6 M guanidine hydrochloride, 5 mM EDTA, 100 mM DTT/DTE, pH 8.5) per 150 mg pellet, followed by incubation for 2 h at room temperature. The pH value was adjusted to approximately 3 by adding 1 M HCl. After centrifugation for 1.5 h at 22,000× *g*, the supernatant was dialyzed in dialysis buffer (4 M guanidine hydrochloride, pH 3). 5 mM TCEP was added and the pH adjusted to 7.6. Centrifugation for 0.5 h at 35,000× *g* and 4 °C was carried out to remove precipitated salt and particles. Thereafter, the volume was doubled with buffer A (4 M GuHCl, 30 mM Tris, 5 mM Imidazole, 2 mM TCEP, pH 7.6). The solution was loaded onto an equilibrated HisTrap FF Crude Column (5 mL, GE Healthcare Life Science, Piscataway, NJ, USA). After a washing step with 25 mM Imidazole in buffer B (4 M GuHCl, 30 mM Tris, 2 mM TCEP, pH 7.6), protein was eluted using 250 mM Imidazole in buffer B. All fractions were collected on ice and then stored at −20 °C. The purification process was evaluated by SDS-PAGE and the protein concentration was determined photometrically by measuring absorbance at 280 nm. The purity of the target protein in the elution fraction was quantified densitometrically on Coomassie brilliant blue-stained SDS gels using open source software ImageJ (version 1.53).

For animal experiments, the elution fraction of the immobilized metal ion chromatography containing HERV-K18(SU) was dialyzed against PBS. The precipitate was then dissolved in buffer C (50 mM Tris, pH 7.4, 150 mM NaCl, 1% SDS, 10 mM DTT) at a concentration of 1 mg/mL. Bacterial endotoxins were determined using the Pierce LAL chromogenic Endotoxin Quantitation Kit (Thermo Fisher Scientific, Waltham, MA, USA) according to the manufacturer instructions and were below 0.5 EU/mL.

### 4.5. SDS-PAGE and Western Blot

Cellular pellets were resuspended in 1x reducing SDS-PAGE loading buffer, boiled at 95 °C for 10 min, and subjected to 12% denaturing SDS-PAGE. Protein samples, which contained GuaHCl, were precipitated using the TCA-DOC/acetone method [51] prior to loading of resuspended protein pellets onto the gel. One of two identical gels was stained with Coomassie brilliant blue G250. The other gel was blotted to a nitrocellulose membrane for Western blot analysis. The membrane was blocked with 5% *w/v* milk powder (MP) in Tris-buffered saline, pH 7.2 (TBS), for 1 h at 24 °C. The membrane was incubated with anti-Penta-His monoclonal antibodies (Qiagen, Hilden, Germany) at a dilution of 1:2000 in 5% MP in TBS at 4 °C overnight, washed three times with TBS containing 0.05% Tween-20 (TBST), and incubated with a 1:2000 dilution of horseradish peroxidase (HRP)-conjugated anti-mouse IgG secondary antibody (Cell Signaling, Cambridge, MA, USA, GB) in 5% MP at 24 °C for 1 h. The membranes were washed three times with TBST and once with TBS, then incubated with SuperSignal West Pico Chemiluminescent Substrate (Thermo Fisher Scientific, Waltham, MA, USA), and His-tagged proteins were detected using the Fusion FX imaging system (Vilber Lourmat, Eberhardzell, Germany).

### 4.6. Mass Spectometry

The protein band corresponding to the target protein was excised from the polyacrylamide gel and destained by repeated incubation in 100 mM NH_4_HCO_3_ (in H_2_O) and 100 mM NH_4_HCO_3_ (in ACN/H_2_O (500/500; *v*/*v*)). Disulfide bridges were reduced with DTT and alkylated with iodoacetamide. Trypsin/Lys-C mix (Promega, V5071) was used for the proteolytic digestion (8 h at 37 °C). Proteolytic peptides were extracted by repeated incubation of the gel band in H_2_O, ACN/H_2_O/TFA (500/450/50; *v*/*v*/*v*), and neat ACN. The obtained extract was concentrated using rotational vacuum concentration and proteolytic peptides were purified with solid-phase extraction using PTFE-based C18 material (Sigma-Aldrich, St. Louis, MO, USA). The eluate was co-crystallized with 2,5-dihydroxybenzoic acid (DHB) on a MALDI ground steel target. MS and MS/MS spectra were acquired using a MALDI-TOF/TOF mass spectrometer (Autoflex Speed, Bruker Daltonics, Billerica, MA, USA) with positive polarity in reflector mode. Data analysis was carried out with PEAKS Studio (version 7.5, Bioinformatics Solutions Inc., Waterloo, ON, Canada) using the human sequences of the UniProt/SwissProt database (release 2019_03), to which the sequence of His6-tagged HERV-K18(SU) was appended. The database search was performed using the following mass tolerances: 50.0 ppm for MS and 0.5 Da for MS/MS.

### 4.7. Mice

Nine to eleven-week-old female mice of the strain SJL/JRj and six week old female mice of the strain Balb/cAnNRj were purchased from Janvier (Le Genest-Saint-Isle, FR). Mice were housed in individually ventilated cages (IVC, Zoonlab, Castrop-Rauxel, DE) with a maximum of five animals per cage (type II, long) under a 12 h light/12 h dark cycle. Water and pelleted food were available ad libitum. Room temperature (22 °C +/−1 °C) and humidity (30–70%) were controlled by a central ventilation system.

### 4.8. Immunization of Balb/cAnNRj and SJL/JRj Mice

All animal experiments were approved by the responsible animal ethics committee of the state of Saxony-Anhalt, Germany (Landesverwaltungsamt Sachsen-Anhalt, Department of Consumer Protection and Veterinary Affairs, Halle (Saale), Saxony-Anhalt, Germany) under the following approval numbers: 42502-2-1271 MLU and 42502-3-851 MLU. We confirm that all animal handling procedures were carried out in accordance with directive 2010/63/EU of the European Parliament and of the Council on the protection of animals used for scientific purposes, the German Animal Protection Act, and recommendations from the Federation of European Laboratory Animal Science Associations (FELASA).

To study the immunogenicity of HERV-K18(SU), Balb/cAnNRj mice (*n* = 5) received daily i.p. injections at days 1–5, 8–12, and 15–19 with 10 µg HERV-K18(SU) in 50 µL buffer D (50 mM Tris, pH 7.4, 150 mM NaCl, 2 M urea, 10 mM DTT) or 50 µL buffer D alone. Blood collection from the retrobulbar venous plexus was performed on days 1, 8, 15, and 22. Another group of mice (*n* = 2–3) received s.c. injections with 200 µL of an emulsion containing either (I) 50 µg recombinant HERV-K18(SU) in buffer E (50 mM Tris, pH 7.4, 150 mM NaCl, 6 M urea, 10 mM DTT) and 100 µL IFA or (II) 100 µL buffer E and 100 µL IFA. On the day of immunization, each mouse was anesthetized by inhalation of anesthesia with 3.5% isoflurane and immunized subcutaneously with 200 μL of freshly prepared emulsion. The total volume was divided into two injections of 100 μL each over the right and left flank. A booster immunization with buffer B in IFA or 50 µg HERV-K18(SU) in IFA was performed 28–30 days after primary immunization. Blood collection from the retrobulbar venous plexus was performed on day 1, on the day of booster, and 9 days after the booster immunization.

To induce experimental autoimmune encephalomyelitis (EAE), 9–11-week-old female SJL/JRj mice were immunized s.c. as described above. A 200 µL emulsion containing 100 µg proteolipid protein (PLP) 139-152(S) (own synthesis) and either (I) Freund’s complete adjuvant (CFA, with 100 µg desiccated *Mycobacterium tuberculosis*), (II) Freund’s incomplete adjuvant (IFA) and buffer C, (III) IFA and 10 µg HERV-K18(SU), or (IV) IFA and 50 µg HERV-K18(SU), respectively, was injected into the mice. After immunization, each mouse was weighted daily and assigned a score according to the severity of the disease (0: no clinical symptoms; 1: limp tail; 2: gait abnormalities; 3: hind limb paralysis; 4: hind limb paralysis plus forelimb paresis; 5: moribund). After 30 days, mice were euthanized using CO_2_ and the brain and spine were removed for histological examinations.

### 4.9. IgG ELISA

HEK293F cells (Invivogen, San Diego, CA, USA) were transfected with pcDNA3.1 (Thermo Fisher Scientific, Waltham, MA, USA) and pcDNA3.1-coK18-C-Flag using Polyethylenimine (PEI, Polysciences, Warrington, PA, USA). Cloning of the expression plasmids is described elsewhere [48]. Protein extraction was performed using a cell extraction buffer (Life Technologies, Carlsbad, CA, USA) according to the manufacturer’s instructions. Then, 96-well MaxiSorp Nunc-immuno plate wells (Thermo Fisher Scientific, Waltham, MA, USA) were coated with 150 µg total protein of cell extracts from transfected HEK293F cells at 15 µg/mL in PBS and incubated overnight at 4 °C. Blocking for 2 h at 4 °C with 200 µL/well Pierce^TM^ protein-free blocking buffer (Thermo Fisher Scientific, Waltham, MA, USA) was followed by three washes with TBS + 0.005% Tween. This was followed by a 2 h incubation with 100 µL plasma samples from immunized Balb/cAnNRj mice (diluted 1:1000 in Pierce^TM^ protein-free T20 blocking buffer (Thermo Fisher Scientific, Waltham, MA, USA)) at 4 °C. After another washing step, bound mouse IgG antibodies were labeled by incubation with goat anti-mouse IgG-HRP (LGC Seracare, Milford, MA, USA) at 1 µg/mL in PBS (100 µL/well) for 1 h at 4 °C. After washing, a color reaction with 1-Step™ Ultra TMB-ELISA substrate solution (Thermo Fisher Scientific, Waltham, MA, USA) was performed and then stopped by the addition of 1.2 N H_2_SO_4_. Absorption at 450/540 nm was determined by a Tecan Sunrise plate reader. For analysis, the OD_450/540_ values of cell extracts from empty-vector transfected cells were subtracted from the OD_450/540_ values of coK18-C-Flag-containing cell extracts. The presence of immobilized target proteins was confirmed using DYKDDDDK tag antibody (1:1000, Cell Signaling, Danvers, MA, USA), followed by secondary antibody goat anti-rabbit-HRP (1:1000, Cell Signaling, Danvers, MA, USA).

### 4.10. T-Cell Proliferation Assay

Spleen harvesting from Balb/c mice was performed 14 weeks after the start of the experiment in i.p. immunized mice. I.p. boosters with 10 µg/mL were given on the 4 consecutive days prior to organ removal. Splenocytes were isolated by repeated flushes of the spleen with PBS. Then, a 10 mL syringe was filled with 10 mL ice cold PBS, and a 26-G cannula was connected to the syringe and plunged into the spleen. By gently pressing the plunger, the cells were flushed out of the spleen capsule. The procedure was repeated two more times. In this way, 1–2 × 10^8^ splenocytes could be collected from a mouse spleen. Splenocytes were seeded in a 96-well microplate at 1 × 10^5^ cells/well in 200 µL cell culture medium (RPMI 1640 + 10% heat inactivated FBS + 1x L-glutamine + 1x Pen/Strep) and stimulated with different amounts of HERV-K18(SU) (0.1–50 µg/mL) for 48 h at 37 °C and 5% CO_2_. Stimulation with 1 × 10^5^ Dynabeads^®^/well (Dynabeads^®^ Mouse T-Activator CD3/CD28, Life Technologies, Carlsbad, CA, USA) served as a positive control and stimulation with buffer D (50 mM Tris, pH 7.4, 150 mM NaCl, 6 M urea, 10 mM DTT) as a negative control. The Cell Proliferation ELISA (Roche, Basel, CH) was performed according to the manufacturer’s instructions with 4 h incubation with BrdU labeling solution. 

The spleens of mice that were immunized s.c. were collected 62–64 days after the start of the experiment. The experimental procedure was the same as described above, except that 2 × 10^7^ splenocytes were subjected to magnetic-assisted cell sorting for isolation of T cells using the Pan T Cell Isolation Kit II, mouse (Miltenyi Biotec, Teterow, Germany, DE), according to the manufacturer’s instructions.

### 4.11. Histology

Brain hemispheres and spines were fixated in 4% paraformaldehyde (PFA, Thermo Fisher Scientific, Waltham, MA, USA) for 24 h or 48 h, respectively. Spines were decalcified in 6% trichloroacetic acid (TCA) solution at 4 °C for 48 h. The tissues were then immersed in 30% sucrose solution for >48 h, embedded using Tissue Tek (Sakura Finetek Europe, Alphen aan den Rijn, The Netherland, NL) and shock-frozen. Then, 10 µm thick frozen sections were prepared using the CryoStarTM NX70 cryostat (Thermo Fisher Scientific, Waltham, MA, USA) and mounted on glass slides. For Luxol fast blue staining, the slides were air-dried, rehydrated for 10 min in PBS, and degreased in 70% ethanol for 24 h, followed by 16 h incubation in a 0.1% Luxol fast blue staining solution at 56 °C. Afterwards, the slides were rinsed two times in distilled water and one time in PBS for 3 min each. The slides were then incubated in a 0.05% aqueous lithium carbonate solution for 3 min before differentiation in 70% ethanol for 1 to 5 min. The differentiation was microscopically monitored and considered complete when the grey matter was discolored and clearly stood out from the (blue colored) white matter. After a Nissl stain with 1% cresyl violet staining solution, the slides were dehydrated in Histo-Clear (National Diagnostics, Atlanta, GA, USA) covered with Permount medium (Thermo Fisher Scientific, Waltham, MA, USA) and analyzed microscopically.

## Data Availability

Data sharing not applicable.

## Supplementary Materials

The following supporting information can be downloaded at: https://www.mdpi.com/article/10.3390/ijms23158330/s1.
