# Supplementary material for: Immunogenicity of the Envelope Surface Unit of Human Endogenous Retrovirus K18 in Mice
Source: Int J Mol Sci. 2022 Jul 28;23(15):8330. doi: 10.3390/ijms23158330 (PMC9369184; doi:10.3390/ijms23158330)
Supplement: Supplementary file 1 [file ijms-23-08330-s001.zip › ijms-1772236-supplementary.pdf]

International Journal of
Molecular Sciences

MDPI

Article

Supplementary material: Immunogenicity of the envelope surface unit of human endogenous retrovirus K18 in mice

Victoria Ilse [1], Rebekka Scholz [1], Michael Wermann [1], Marcel Naumann [1], Martin S. Staege [2], Steffen Roßner [3] and Holger Cynis [1,*]

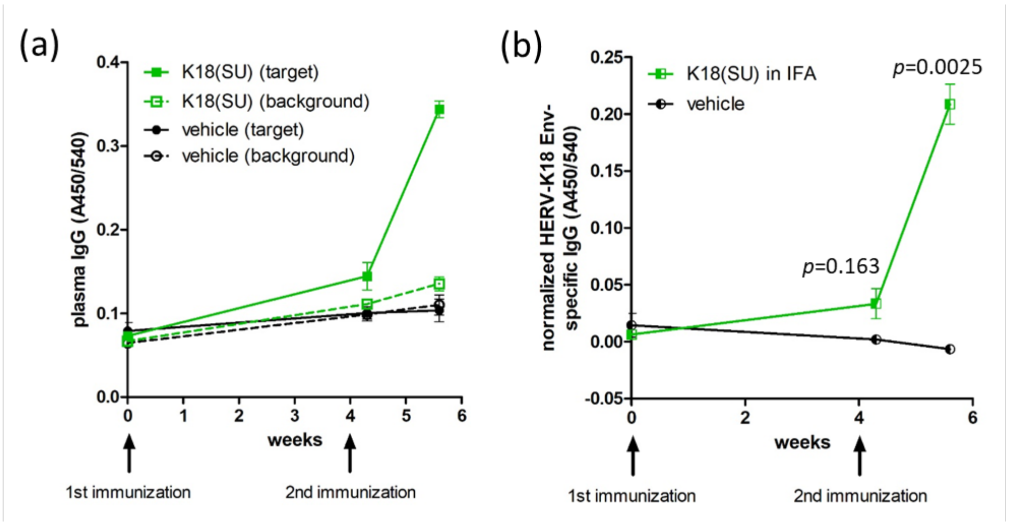

Figure S1. Antibody response to recombinant HERV-K18(SU), second experiment. (a) and (b) 6 weeks old female Balb/c mice received two subcutaneous immunizations with 50 µg HERV-K18(SU) (n=3) or vehicle (n=2) in IFA at a four-week interval. Plasma IgG titers were analyzed at three time points using HERV-K18 Env-specific ELISA. Because ELISA plates could not be coated with insoluble recombinant HERV-K18(SU), cell extracts that did (target) or did not (background) contain HERV-K18 Env were used instead. Graphs in (a) and (b) show original absorbance data and the background subtracted values, respectively. Data of the second of two experiments is shown as mean ± SEM. Statistics: unpaired t-test (K18(SU) versus vehicle).
